# Lymphocyte activation gene 3 in autoimmune disease: from molecular mechanisms to clinical applications

**DOI:** 10.3389/fimmu.2026.1904328

**Published:** 2026-08-18

**Authors:** Lishuang Guo, Lu Pan, Congcong Liu, Sirui Yang

**Affiliations:** 1 Department of Pediatric Rheumatology, Immunology & Allergy, Children’s Medical Center, The First Hospital of Jilin University, Changchun, China; 2 The Child Health Clinical Research Center of Jilin Province, Changchun, China

**Keywords:** autoimmune disease, inhibitory immune receptors, LAG3, mechanism, molecular mechanism

## Abstract

Lymphocyte activation gene 3 (LAG3) has become an important immune checkpoint receptor, which plays a crucial role in regulating the immune response. The immunoregulatory function of LAG3 is achieved through complex receptor-ligand interactions, structure-function relationships, and unique signaling pathways. The role of LAG3 in cancers has been widely explored, and drugs targeting LAG3 are constantly emerging, demonstrating promising application prospects. In autoimmune diseases, downregulation of LAG3 leads to the breakdown of immune tolerance and excessive immune activation. In this review, we begin with the mechanisms by which LAG3 exerts its immunosuppressive function and its role in the immune system, focusing on the role of LAG3 in autoimmune diseases and recent research progress.

## 1 Introduction

Inhibitory immune receptors (IRs), also known as immune checkpoints, are a group of cell surface receptors with negative regulatory functions. They limit immunopathological responses by regulating the functions of immune cells and promote self-tolerance, playing a key role in regulating immune responses (1, 2). More than 300 potential IRs are encoded in the human genome, although only a few have been identified, including cytotoxic T-lymphocyte-associated protein 4 (CTLA-4), programmed cell death protein 1 (PD-1) and T cell immunoglobulin and mucin domain containing protein-3 (TIM3) (3). In tumors and infectious diseases, these molecules are upregulated as a mechanism of immune escape. Conversely, the absence of inhibitory immune checkpoint signaling can lead to the development of autoimmune diseases (AIDs) (4). Numerous studies have reported abnormal expression, dysregulated function, and impaired immune regulation of IRs in AIDs, emphasizing their important role in the pathogenesis of these disorders (**Table 1**). Furthermore, immune checkpoint inhibitors can induce a series of autoimmune-like inflammatory conditions affecting multiple organs during cancer therapy, known as immune-related adverse events (5), which further supports this concept. The expression of IRs and their ligands vary across tissues, cell types, and cell subsets and changes according to the activation status of cells. Consequently, different IRs fine-tune immune responses in a highly specific manner.

**Table 1:** The role of inhibitory receptors in autoimmune disease.

IRs	Ligands of IRs	Expressing cells	Mechanism of inhibition	Mechanisms associated with AIDs	Related AIDs	References
CTLA-4	CD80/CD86	Activated T cells, Tregs, exhausted effector T cells	1) Inhibition of TCR and CD28 proximal signals 2) CTLA-4 with CD28 competing CD80/CD86 on activated antigen-presenting cells 3) By reducing the interaction time between APC and T cells, the activation of T cells is reduced 4) Secreting cytokine IL-10, TGF-β reduces the expression or availability of CD80/CD86. 5) Inhibitory signals into APC via retrograde delivery of ligands	Decreased CTLA-4 expression disrupts CD28 co-stimulation, weakens Treg suppression, expands autoreactive T cells and increases autoantibody secretion by B cells. Autoreactive lymphocytes then infiltrate organs, causing inflammatory tissue injury and progressive autoimmune disorders.	RA, SLE, MS, T1D, MG	(134–136)
PD1	PD-L1/PD-L2	Activated T cells, Tregs, exhausted effector T cells, NK, B cells, macrophages	1) Recruits the phosphatase SHP2 via the intracellular ITSM motif 2) Inhibits PI3K/Akt and ERK pathways, reduces pro-inflammatory cytokines like IL-2 and IFN-γ 3) Suppresses CD28 and, to lesser extent TCR signaling 4) Prevents follicular recruitment of T cells	Loss of PD-1 lets autoreactive T cells circulate widely; impaired PD-1/PD-L1 axis unleashes autoreactive lymphocytes, breaks immune tolerance and triggers AIDs.	SLE, MS, T1D, EAE, AITD, RA, GD, SS	(137–140)
TIGIT	CD112/CD155	Activated T cells, Tregs, Tr1, Tfh, memory T, NK, NKT, exhausted effector T cells	1) IGIT interacts with CD155 to elicit direct inhibitory signals in T/NK cells 2) Engagement of CD155 on DCs by TIGIT induces immunosuppressive DCs by triggering IL-10 production while decreasing IL-12 secretion and CD86 expression, which indirectly inhibit T cells function 3) Activation of TIGIT enhances Treg-mediated suppression via secretion of IL-10, suppression of Th1/Th17, and inhibition of Th1 programs 4) Disrupts CD226 homodimerization to impede CD226-mediated stimulatory signals	TIGIT deficiency relieves suppression of effector T/NK cells, impairs tolerogenic DCs and Tregs, removes CD226 inhibition, and boosts autoantibody and proinflammatory cytokine secretion to worsen tissue injury.	SLE, AIH, RA, MS, PSO, IBD, T1D, SS, EAE	(141, 142)
CD200	CD200R1-5	T cells, macrophages, neutrophils, Myeloid cells, DCs, mast cells, Basophils, NK, MDSC	1) Membrane CD200 cross-links CD200R1 and induces tyrosine phosphorylation of CD200R within the NpxY (286/297) motifs, thereby inhibiting mast cells and myeloid cells 2) CD200R ligation also stimulates IDO expression in plasmacytoid DCs, mimicking the effects of B7/CTLA4 signaling, thus reinforcing the tolerogenic properties of some DC subsets 3) downstream augmentation of TGFβ (and IL-10) production	The role of CD200 in AIDs remains unclear. In SLE, CD200R shifts to a pro-inflammatory receptor under type I IFN stimulation, exacerbating autoinflammation. Activation of CD200 alleviates disease in EAU and RA, while it exerts only mild effects in SS.	CIA, RA, SLE, SS, EAU	(143, 144)
CD22	Sialylated surface protein	B cells (especially mature B cells), cDC, mast cells	CD22 inhibits B cell receptor signaling through immunoreceptor tyrosine heterogeneous motif (ITIM), and plays a role in maintaining humoral immune homeostasis	Upon CD22 inactivation, nucleic acid immune complexes continuously activate TLR7 and trigger massive type I interferon secretion, causing multi-organ immune complex-mediated injury. Impaired CD22 function leads to overactivation of autoreactive B cells and elevated autoantibodies.	SLE, RA, TTP, GD, EAE	(145, 146)
BDCA2	Asialo-galactosyl oligosaccharide	pDC	BDCA2 signals through an associated transmembrane adaptor, the FcϵRγ, which recruits the protein tyrosine kinase Syk, inducing protein tyrosine phosphorylation and calcium mobilization, which reduces TLR-induced activation of pDC, inhibiting type I IFN secretion and other inflammatory mediators	Defective BDCA2 signaling leads to sustained IFN-α secretion by pDCs, which drives autoimmune inflammation and AIDs.	SLE, SSc, PSO	(147, 148)
VSTM1	Gal-1	Myeloid-derived APCs, T cells, Tregs	1) Activation of VSTM1 preserved neutrophil viability by suppressing ROS production 2) Overexpression of VSTM1 on monocytes has been shown to inhibit cell proliferation, chemotaxis, and proinflammatory cytokine production	Dysregulated VSTM signaling triggers neutrophil oxidative stress and massive necrosis with self-antigen release, driving autoantibody production and multi-organ inflammatory damage.	SLE, RA	(149, 150)
PILRα	Sialylated surface protein	Myeloid cells, macrophages, DCs granulocytes	1) Negatively regulates neutrophil infiltration via modulation of integrin activation during inflammation 2) Controls monocyte mobility through regulating integrin signaling and inhibiting CD99–CD99 binding 3) Interact with CD8A and keep CD8 T cell in a quiescent state, thus maintaining the T cell pool size in the absence of antigen exposure	PILRα deletion enhances proinflammatory cytokine secretion via the ITIM-SHP pathway, exacerbating synovial hyperplasia and bone destruction.	RA	(151, 152)
LAG3	MHC-II/FGL1/LSECtin/α-synuclein	Activated T cells, Tregs, Tr1, NK, NKT, B cells, plasma cells, pDCs, exhausted effector T cells, neurons	1) LAG3 cross-linking induces down-regulation of CD3/TCR complex expression, inhibits TCR-induced calcium fluxes and finally results in functional unresponsiveness of T cells 2) Mediate negative signaling into the interacting APCs	LAG3 exhibits dual properties in AIDs. Classically, LAG3 deficiency disrupts immune tolerance and initiates AIDs. On the other hand, persistent self-antigen stimulation drives substantial upregulation of LAG3 on pathogenic effector T cells; under such circumstances, the inhibitory capacity of LAG3 fails to counteract the proinflammatory activity of these cells, resulting in AIDs such as psoriasis.	T1D, IBD, MS, RA, PSO	(5)
TIM3	Gal-9/CEACAM-1/HMGB-1/PtdSer	Activated T cells, Tregs, Th17, DCs, NK, B cells, exhausted effector T cells, monocytes	1) Induce the apoptosis of T helper cells and weaken the activation and differentiation of other immune cells 2) Binding to ligands leads to T cell exhausted 3) Treg cells with high expression of TIM3 secrete IL-10 and inhibit the function of effector T cells	Impaired TIM-3 signaling disrupts self-tolerance and aggravates tissue inflammation, while TIM-3/Gal-9 activation relieves autoimmunity; Its function is cell- and disease stage-dependent with dual regulatory effects.	RA, CIA, EAE, SLE, MS, RA, IBD, CD	(153, 154)
B7H3	unknown	Activated T cells, NK, DCs, monocytes, macrophages, neutrophils, MDSC, B cells	1) Reports of both costimulatory (T cells) and coinhibitory (T cells and NK cells) functions 2) Role in osteoclast differentiation and bone mineralization	B7-H3 has dual immunomodulatory functions: soluble B7-H3 exerts anti-inflammatory protective effects, while membrane B7-H3 fuels Th1/Th17 inflammation and tissue damage in RA, MS and SS; conversely, it inhibits autoantibodies and mitigates renal injury in SLE.	AITD, GD, RA, MS, AS, T1D, SS	(155–158)
BTLA	HVEM	B cells, T cells, Tregs, NK	1) Intracellular ITIM motifs get phosphorylated and recruit SHP-1/SHP2 phosphatases, then inhibits TCR, BCR and TLR signaling 2) Reduces secretion of IL-2, IFN-γ, IL-17 and other pro-inflammatory cytokines.	1) Impaired BTLA-HVEM pathway leads to excessive activation of Th1/Th17, Tfh and autoreactive B cells, thereby inducing tissue injury. 2) It can also bind LIGHT to trigger pro-inflammatory signals; the binding mode determines the final immune outcome.	ITP, RA, SLE, SSc, T1D, MS, IBD, PSO	(159, 160)

TTP, thrombotic thrombocytopenic purpura; SLE, systemic lupus erythematosus; RA, rheumatoid arthritis; T1D, type 1 diabetes; MS, multiple sclerosis; MG, myasthenia gravis; EAE, experimental autoimmune encephalomyelitis; AITD, autoimmune thyroid disease; SS, Sjögren’s syndrome; PSO, Psoriasis; IBD, Inflammatory bowel diseases; GD, Graves’ disease; CIA, Collagen-induced arthritis; AS, ankylosing spondylitis; TIP, immune thrombocytopenia; SSc, systemic sclerosis; CD, Crohn’s Disease; EAU, experimental autoimmune uveoretinitis; CTLA-4, cytotoxic T lymphocyte antigen 4; PD-1, programmed cell-death-1; PD-L1, programmed death ligand-1; TIM3, T cell immunoglobulin and mucin domain containing protein-3; TIGIT, T cell immunoreceptor with Ig and ITIM domains; BTLA, B and T lymphocyte attenuator; HMGB-1, High mobility group protein B-1; PtdSer, Phosphatidylserine; Gal, Galectin; CEACAM1, Carcinoembryonic Antigen-Related cell adhesion molecule 1; VSTM1, V-set and transmembrane domain-containing 1; BDCA2, Antibody-binding of blood dendritic cell antigen 2; HVEM, herpesvirus entry mediator; PILRα, paired immunoglobulin-like type 2 receptor alpha; LAG3, Lymphocyte activation gene-3; MHC-II, major histocompatibility complex II; FGL1, Fibrinogen-like protein 1; LSECtin, Liver sinusoidal endothelial cell lectin; APC, antigen-presenting cells; NK, natural killer cells; NKT, natural killer T cells; Tfh, follicular helper T cell; Treg, regulatory T cell; Th, T helper cells; Tr1, type 1 regulatory T cells; MDSC, Myeloid-derived suppressive cells; IDO, indoleamine-2, 3-dioxygenase.

Lymphocyte activation gene 3 (LAG3), also known as CD223, is a member of the inhibitory IRs and is widely expressed in various immune cells, including CD4^+^ T cells, CD8^+^ T cells, natural killer (NK) cells, and regulatory T cells (Tregs) (**Table 1**) (6). Upon stimulation by factors such as antigens, LAG3 expression is upregulated (7), thereby negatively regulating T-cellproliferation, activation, effector function and homeostasis (8). The unique signaling patterns and diverse ligands of LAG3 suggest that it may exert broader and more complex inhibitory effects than CTLA-4 and PD-1 (9). To date, most studies on LAG3 have focused on its immunoinhibitory roles in tumors and chronic viral infections. In recent years, increasing evidence has indicated that LAG3, either alone or in conjunction with other IRs, coordinately regulates autoimmunity and prevents excessive immune responses (10). Studies have shown that, in mice with susceptible genetic backgrounds, the absence of LAG3 can lead to the development of AIDs (11), and the combined deficiency of LAG3 and PD-1 can result in fatal autoimmune myocarditis (12). Meanwhile, LAG3 overexpression can effectively alleviate disease symptoms in mouse models of AIDs (13). Collectively, this evidence supports the the involvement of LAG3 in the development of AIDs. In this review, we first introduce the structure, ligands, and mechanism of action of LAG3. We then summarize the effects of LAG3 on the immune system and its involvement in various AIDs. Finally, we discuss the progress of LAG3 agonists in the treatment of AIDs.

## 2 The characteristics of LAG3

### 2.1 Structure of LAG3

**Figure 1:**
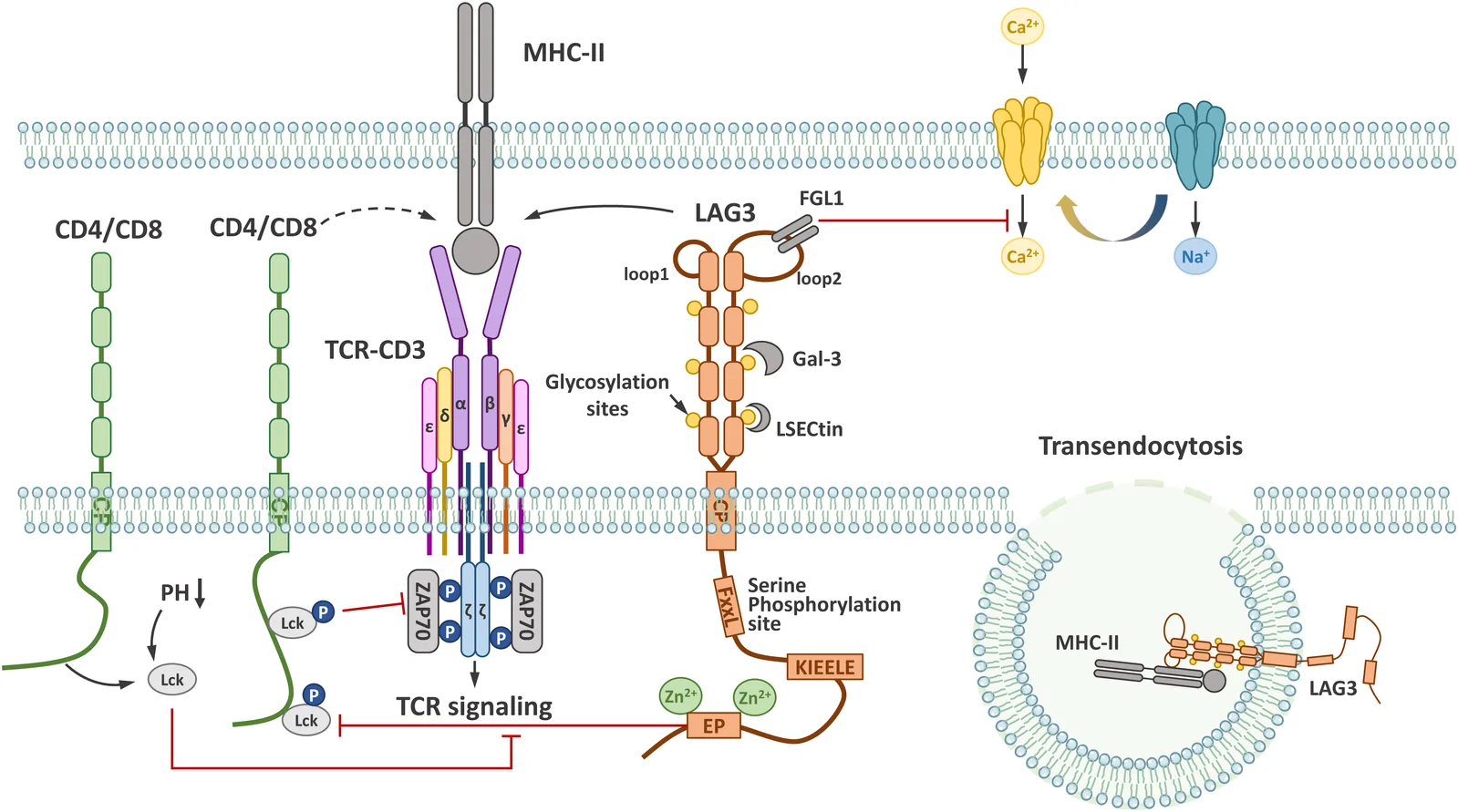
The structure and inhibitory mechanism of LAG3. The interaction between LAG3 and MHC-II can recruit LAG3 to the TCR-CD3 complex, attenuates the binding of CD4 to MHC-II, and promote the endocytosis process of MHC-II into the T cell. After the two combines, their intracellular domains will reduce the local pH value around the TCR-CD3 complex, acidify the intracellular environment. This triggers the dissociation of the TCR-activating kinase Lck from CD4 and CD8 co-receptors, thereby exerting ligand-independent inhibitory effects. Meanwhile, MHC-II binds to Zn^2+^, thereby interfering with the interaction between the co-receptor and Lck, and subsequently inhibiting the phosphorylation of CD3ζ and ZAP70 as well as the downstream TCR signal transduction. Moreover, the simultaneous binding of LAG3/TCR to its ligand will also inhibit the intracellular calcium ion flow dependent on TCR/CD3.

In 1990, Triebel et al. (14) first identified LAG3 as a novel lymphocyte activation gene located at the distal end of the short arm of human chromosome 12 (12p13.32) and mouse chromosome 6, adjacent to CD4, with which it shares high structural homology. LAG3 encodes a type I transmembrane protein that belongs to the Immunoglobulin (Ig) superfamily. This protein consists of an extracellular region, a transmembrane region, and an intracellular region (**Figure 1**). The extracellular region comprises four Ig superfamily domains (D1-D4), including eight cysteine residues, and four N-linked glycosylation sites. Among these domains, D1 is a variable-like V-type domain. Within the D1 structure is a proline-rich loop, termed loop1, which was previously considered the binding site for major histocompatibility complex class (MHC-II). In addition, some studies have suggested the presence of a second loop, termed loop2, on the side of D1 opposite the MHC-II-binding site, which serves as the binding site for fibrinogen-like protein 1 (FGL1) and LAG3 (15). Domains D2-D4 are C2-type domains. Recent studies have shown that, unlike the monomeric structure of CD4, both human LAG3 (hLAG3) and mouse LAG3 (mLAG3) crystallize as parallel homodimers. Dimerization is primarily mediated by hydrophobic interactions at the D2-D2 interface, and the mLAG3 dimer appears to be more stable than the hLAG3 dimer (15, 16). The transmembrane linker peptide adjacent to D4, which is longer than that of CD4, is readily cleaved by a disintegrin and metalloproteinase, resulting in the generation of soluble LAG3 (sLAG3) (17). The biological function of sLAG3 remains controversial and is largely unknown. Some studies have shown that sLAG3 binds to MHC-II to promote Dendritic cells (DCs) maturation and provide costimulatory signals. However, these findings are primarily derived from synthetic high-affinity sLAG3-Ig fusion proteins, which differ substantially from endogenous human sLAG3 (18). Human sLAG3 has been suggested to inhibit monocyte differentiation, impair antigen (Ag) presentation, and suppress T-cell proliferation and activation, thereby exerting coinhibitory immune effects (19). Although the biological function of sLAG3 has not been fully elucidated, it has emerged as a predictive biomarker for many malignant tumors. In multiple solid tumors, including lung cancer, elevated sLAG3 levels correlate with shorter progression-free survival (PFS) and overall survival (OS), serving as an independent adverse prognostic factor (20). A recent study has shown that sLAG3, together with other soluble immune markers, can serve as a liquid biopsy tool for the subtyping and prognostic evaluation of neuroendocrine neoplasms (21). To date, research on the role of sLAG3 in AIDs remains limited. Elevated plasma sLAG3 levels have been detected in patients with active immune thrombocytopenia (ITP) (22). Similar findings have also been reported in patients with Rheumatoid Arthritis (RA). However, no significant difference was observed between patients with RA in remission and those with moderate to severe disease activity (23). The interactions of sLAG3 with various cytokines and immune cells in chronic viral infections suggest that it plays a critical immunoregulatory role (24, 25).

The cytoplasmic tail of LAG3 consists of three conserved motifs: (1) a putative serine phosphorylation site that serves as a substrate for protein kinase C, S484/FxxL motif; (2) the “KIEELE” motif, whose lysine residue is crucial for its immunosuppressive function and which may act either independently or in conjunction with the other two conserved motifs; (3) The “EP” motif, formed by repeated glutamic acidproline dipeptides. The latter motif binds to LAG3-associated proteins and was shown in early studies to be involved in the downregulation of the CD3/T-cell receptor (TCR) activation pathway (26). Furthermore, several studies have shown that mutations in the EP motifcontribute to maintaining the activity and function of LAG3 (27). This observation has led to the proposal that the EP motif may prevent LAG3 from acting as a stimulatory coreceptor, rather than cooperating with the KIEELE motif to inhibit signal transduction. Therefore, the EP motif is not required for the inhibitory function of LAG3.

### 2.2 Ligands of LAG3

#### 2.2.1 MHC class II molecules

LAG3 was first shown to inhibit T-cell activation through engagement with its canonical ligand, MHC-II. Despite sharing structural homology with CD4 and exhibiting a higher affinity for MHC-II, LAG3 does not competitively inhibit CD4 binding. Instead, LAG3 preferentially suppresses T cells responsive to stable peptide-MHC-II (pMHC-II) complex by transducing inhibitory signals through its intracellular domain (28). This finding indicates that the inhibitory activity of LAG3 preferentially targets peptides with high affinity for MHC-II. A recent study validated this hypothesis and demonstrated that the spatial proximity of LAG3 to the TCR facilitated by cognate pMHC-II complexes, is critical for the suppression of CD4^+^ T cells (29). The binding mode of LAG3 to MHC-II has long been the subject of debate. Loop1 was previously considered the MHC-II-binding site. However, recent structural studies have revealed that the BC loop, C′ strand, C′C″D loop, and DE loop within the D1 domain of hLAG3 together form a discontinuous binding interface for human leukocyte antigen class-II (30). In mLAG3, the binding interface is centered on the loop 2 region connecting the C′ and D strands of D1, with additional contacts formed by an adjacent patch of charge-complementary residues (31). Although loop1 does not directly interact with MHC-II, deletion of loop1 reduces the binding affinity between LAG3 and MHC-II. These findings indicate that loop1 may indirectly facilitate this interaction by promoting dimerization or stabilizing the binding conformation of the D1–D2 domains (32).

#### 2.2.2 Fibrinogen-like protein 1

FGL1, a member of the fibrinogen family, is a liver-derived protein involved in hepatic secretion, proliferation, and metabolism (33). In 2019, FGL1 was identified as a novel high-affinity ligand for LAG3 that functions independently of MHC-II. FGL1 binds to the D1 domain of LAG3 through a flexible loop within its C-terminal fibrinogen-like domain (15). In mice, FGL1 deficiency results in the delayed onset of autoimmune symptoms (34), and FGL1 exerts anti-inflammatory effects and demonstrates therapeutic efficacy in murine models of collagen-induced arthritis (CIA) (35). These findings indicate that FGL1 plays a critical role in maintaining immune homeostasis. *In vitro*, the addition of recombinant FGL1 protein partially inhibits T-cell activation in a LAG3-dependent manner (36). Previous studies have demonstrated that the FGL1/LAG3 axis constitutes an independent immunosuppressive pathway. The interaction between LAG3 and FGL1 enhances the suppression of T-cell activation and inflammatory cytokine secretion (37). Furthermore, FGL1 directly inhibits LAG3-expressing hepatic T cells and NK cells (38). Conversely, some studies suggested that LAG3-mediated T-cell inhibition is independent of FGL1 (39). Therefore, the immunosuppressive role of FGL1 as a ligand of LAG3 remains to be confirmed by further investigation.

#### 2.2.3 Other ligands

Recent studies have indicated that the T cell TCR-CD3 complex acts as a cis ligand for LAG3. Unlike MHC-II, both the TCR-CD3 complex and LAG3 are expressed on the T-cell surface, where they have recently been shown to engage in cis interactions. Guy et al. (40) found that LAG3 translocate to the immunological synapse and associates with the TCR-CD3 complex in CD4^+^ and CD8^+^ T cells, even in the absence of MHC-II binding. However, this finding does not preclude a role for MHC-II, which may be critical for amplifying LAG3 function when LAG3 expression levels on T cells are limited. The immunosuppressive mechanism by which the TCR-LAG3 complex functions as a LAG3 cis ligand is discussed in the section entitled “Inhibitory mechanism of LAG3.”

Galectin-3 (Gal-3) belongs to the galectin family and is a potential ligand of LAG3. Gal-3 mediates multiple key intracellular signaling pathways, thereby promoting cytokine release and playing critical roles in inflammation, immune regulation, and tumorigenesis (41). In mice, Gal-3 accelerates the development of autoimmune cholangitis by driving NLRP3 inflammasome activation and the consequent production of interleukin-1β (IL-1β) (42). *In vitro*, T cells cultured with Gal-3 exhibit reduced production of proinflammatory cytokines (43). Gal-3-mediated inhibition of CD8^+^ T cells is dependent on LAG3 expression (44).

Liver sinusoidal endothelial cell lectin (LSECtin), a member of the C-type lectin receptor family, has been identified as another potential ligand of LAG3 (45). In melanoma cells, LSECtin interacts with the coregulatory molecule LAG3, and blockade of this interaction restores interferon-γ (IFN-γ) secretion, which is impaired by LSECtin expression (46). However, current evidence supporting Gal-3 and LSECtin as ligands of LAG3 remains limited and requires further validation.

The propagation of pathological α-synuclein (α-syn) is increasingly recognized as an important driver of Parkinson’s disease (PD). LAG3 binds to α-syn preformed fibrils (PFFs), triggering their endocytosis, toxicity, and neuron-to-neuron transmission, thereby inducing pathological changes in mouse models of PD (47). A subsequent study demonstrated that the D1 domain of LAG3 uses a positively charged surface to bind the acidic C-terminus, of α-syn, particularly residues 118-140. Furthermore, phosphorylation at serine 129, a hallmark modification of pathological α-syn, further enhances the interaction between α-syn fibrils and LAG3 (48). More recently, LAG3 knockout (KO) has been shown to suppress the aggregation of α-syn PFFs, thereby blocking the pathogenic T-cell responses induced by α-syn PFFs (49). These findings provide a scientific basis for the development of therapeutic strategies targeting the interaction between α-syn fibrils and LAG3 in PD.

### 2.3 Inhibitory mechanism of LAG3

The inhibitory signaling mechanism of LAG3 has not been fully elucidated. Upon ligand binding, LAG3 negatively regulates T-cell function through its intracellular domain. Notably, LAG3 lacks canonical signaling motifs, such as immunoreceptor tyrosine-based inhibitory motifs and immunoreceptor tyrosine-based switch motifs, which are well characterized in other inhibitory receptors. A previous study found that deletion of the KIEELE motif completely abrogated the inhibitory function of LAG3 in CD4^+^ T cells (50). The FxxL motif is located in the membrane-proximal region of the intracellular domain. Substitution of either phenylalanine (F475) or leucine (L478) with alanine markedly attenuates the inhibitory activity of LAG3 (51). Louzalen et al. (26) proposed that the EP motif is critical for counteracting the CD3/TCR activation pathway. However, a recent study demonstrated that LAG3 transduces two independent inhibitory signals: one through the membrane-proximal FxxL motif and the other though a C-terminal EP repeat (51). These motifs are the primary mediators through which LAG3 modulates TCR signaling. Downstream, LAG3 disrupts the interaction between the CD4/CD8 coreceptors and the tyrosine kinase Lck, thereby limiting the phosphorylation of CD3ζ and ZAP70 and ultimately suppressing the TCR signaling cascade (40). A recent study further demonstrated that the proximity of the TCR plays a critical role in MHC-II-dependent suppression of CD4+ T cells by LAG3 through disruption of the intracellular association between CD3ϵ and Lck (29). It should be noted that LAG3 dimerization may contribute to increased ligand-binding avidity. Evidence has indicated that forced dimerization of sLAG3 through an Fc tag significantly enhances its binding to MHC-II (52). A mutagenesis study showed that dimerization-disrupting mutations weaken the binding of mLAG3 to FGL1 and MHC-II and abolish their inhibitory effects on T-cell activation *in vitro* (16). Additionally, the inhibitory function of LAG3 is closely associated with its cell surface expression level: the higher the expression of LAG3, the greater the impaired of T-cell effector function (53).

LAG3 exerts inhibitory effects even in the absence of ligand binding. LAG3 was recently found to induce trans-endocytosis of MHC-II into both CD4^+^ and CD8^+^ T cells (**Figure 1**). This process reduces the Ag-presenting capacity of antigen-presenting cells (54). Imaging studies have revealed that LAG3 colocalizes with the TCR at the immunological synapse, and co-immunoprecipitation studies have further demonstrated its association with the TCR/CD3 complex. As previously mentioned, ligand-independent inhibition by LAG3 appears to occur through LAG3-mediated dissociation of Lck from T cells (**Figure 1**) (30). Multiple studies have also confirmed that dual KO of PD-1 and LAG3 in CD8^+^ T cells markedly enhance antitumor immunity (55), further highlighting that LAG3 can suppress CD8^+^ T cells that do not normally interact with MHC-II. Collectively, these findings highlight the complexity of LAG3-mediated immune regulation and its underlying molecular mechanisms.

## 3 The function of LAG3 in immune system

### 3.1 LAG3 on adaptive immune cells

#### 3.1.1 T cell subpopulations

LAG3 is expressed in most activated T cell populations, including CD4^+^ T cells and CD8^+^ T cells (28). In T cells, LAG3 expression is induced upon TCR stimulation or by cytokines such as IL-12, IL-2, IL-15, IL-6, IL-7, IL-8, and interferon-β (56). A previous study demonstrated that CD25 expression was elevated in the presence of an anti-LAG3 monoclonal antibody (mAb), accompanied by increased levels of IL-4 and IFN-γ, supporting the negative regulatory role of LAG3 in CD4^+^ T cells (57). *In vivo*, LAG3^-/-^ T cells exhibited delayed cell cycle arrest following stimulation with the superantigen staphylococcal enterotoxin B, resulting in enhanced T-cell expansion and splenomegaly (58). The same study also reported that infection of LAG3^-/-^ mice with Sendai virus increased the numbers of memory CD4^+^ and CD8^+^ T cells (58). In a mouse model of E. multilocularis infection, LAG3 expression was predominantly detected in T helper 2 (Th2) and Treg subsets, which secreted significantly higher levels of IL-4 and IL-10. Furthermore, LAG3 deficiency promoted the development of effector memory CD4^+^ T cells and enhanced the type 1 CD4^+^ T-cell immune response (59).

Notably, LAG3 is expressed on CD8^+^ T cells in a variety of tumors and has been associated with the prognosis of solid tumors (60). In many tumors, LAG3 expression is elevated on CD8^+^ tumor-infiltrating lymphocytes, accompanied by impaired effector function (61–63). In recent years, the co-expression of LAG3 and PD-1 has been found to enhance the inhibition of CD8^+^ T cells. Blockade of both PD-1 and LAG3 on CD8^+^ T cells promotes their tumoricidal activity (64). Previous clinical trials have shown that nivolumab (an anti-PD-1mAb) combined with relatlimab (an anti-LAG3 mAb) improves PFS and OS in patients with advanced melanoma compared with nivolumab monotherapy (65, 66). This combination regimen was approved by the U.S. Food and Drug Administration for the treatment of advanced melanoma in early 2022. Recently, several mechanistic studies have provided further insights. Andrews et al. reported that deletion of PD-1 and LAG3 increased interferon-responsive gene expression and IFN-γ secretion (67). Another study demonstrated that LAG3 deficiency enhanced the cytotoxic activity of CD8^+^ T cells through the CD94/NKG2-Qa-1b axis (55). A clinical analysis of melanoma tissue samples showed that relatlimab and nivolumab enhanced CD8^+^ TCR signaling and altered CD8^+^ T cell differentiation, thereby enhancing cytotoxicity while maintaining an exhausted phenotype (68). Furthermore, the combination of nivolumab and relatlimab has shown promising efficacy in gastrointestinal tumors and B-cell malignancies (69–71).

**Figure 2:**
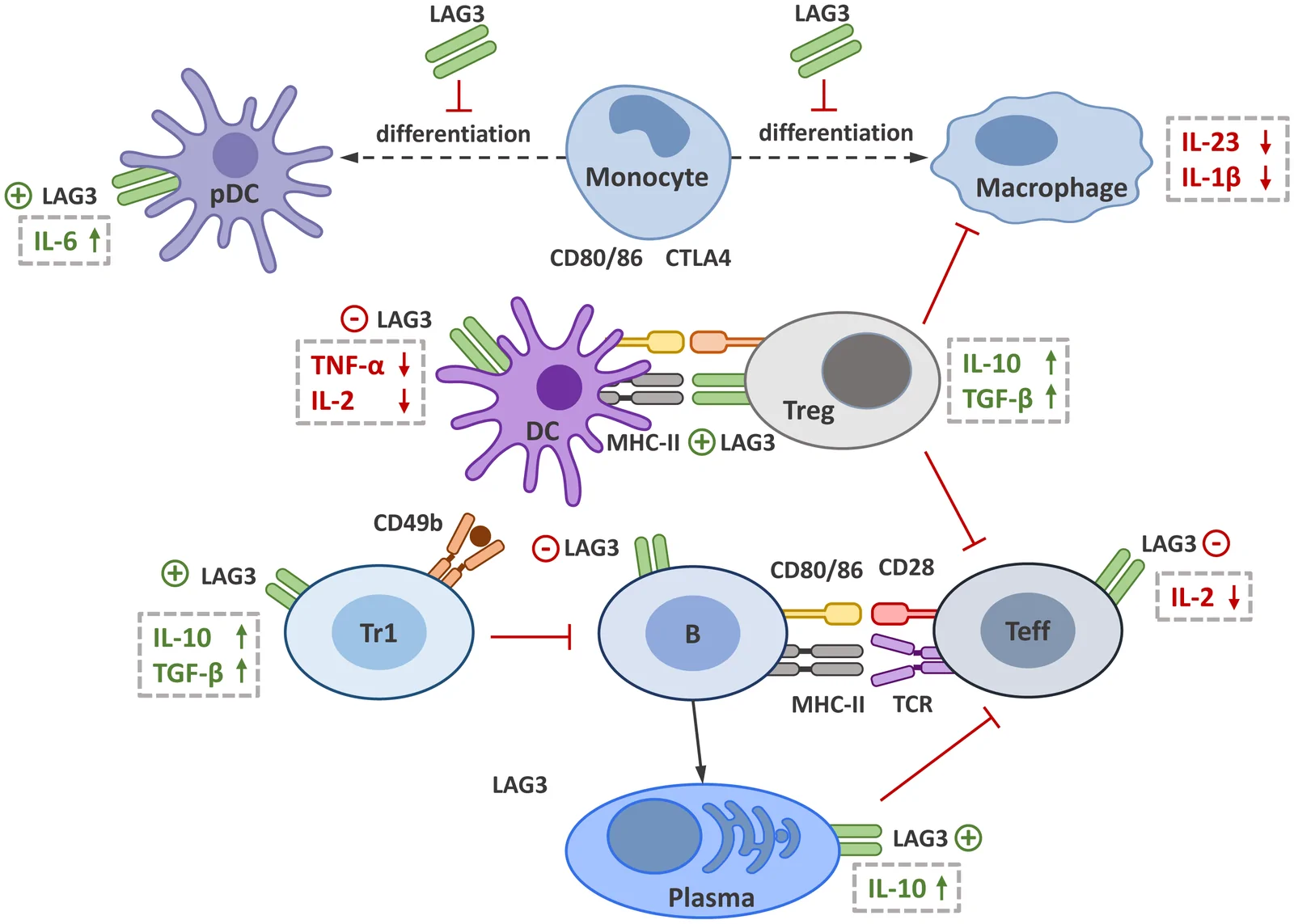
The role of LAG3 in the immune system. LAG3 regulate immune responses through various mechanisms, including: 1) the expression of LAG3 reduced the differentiation of monocytes into macrophages and DCs. 2) LAG3 on pDCs increased the secretion of IL-6, and decreased the secretion of IL-2 and TNF-α by DCs. 3) The expression of LAG3 prompts Treg, including Tr1 cells, to produce heterogeneous factors such as IL-10 and TGF-β, while inhibiting the response of DCs. 4) The upregulated LAG3 promotes the secretion of IL-10 by plasma cells, thereby inhibiting the function of Teff.

Tregs mediate tolerance to self-antigens and maintain immune homeostasis. LAG3 is a key molecule required for Tregs to exert their maximal suppressive function (72). Previous studies have suggested that LAG3 limits the proliferation and function of Tregs (73). In a mouse model of Echinococcus multilocularis infection, LAG3-KO CD4^+^ T cells were more likely to differentiate into T helper 1 (Th1) cells and less likely to develop into Tregs (59). However, in atherosclerotic mice, LAG3 deficiency increased the frequencies of both Th1 and effector/memory T cells, while Treg proportions were concomitantly elevated, resulting in a counterbalancing immunoregulatory effect (74). Therefore, the regulatory effect of LAG3 on Treg differentiation remains to be further elucidated. Mechanistically, Treg cells reduced IL-22 production by binding to MHC-II on CX3CR1^+^ macrophages through LAG3 (75). A recent study indicated that LAG3 enhanced Treg-mediated suppression partly through modulation of Myc-dependent metabolic programming (76). Numerous studies have highlighted that LAG3^+^ Tregs exert suppressive effects through IL-10 (**Figure 2**), one of the major immunosuppressive cytokines (77). A specialized Treg subset that secretes IL-10 and transforming growth factor-beta (TGF-β) but does not express Foxp3 is designated as type 1 regulatory T (Tr1) cells. These cells co-express CD49b and LAG3 (78). Beyond serving as a marker of Tr1 cells, LAG3 plays a limited role in Tr1-mediated suppression. This observation is consistent with findings in Foxp3^+^ Tregs, which exhibit enhanced suppressive capacity when expressing LAG3. Tr1 cells inhibit the production of pathogenic autoantibodies by autoreactive B cells and promote their elimination (79).

#### 3.1.2 B cell subpopulations

Splenic B cells, peritoneal B‐1a cells, and Peyer’s patch B cells can give rise to a distinct regulatory B cell (Breg or B10^+^) subset with immunosuppressive functions, including IL-10 production and LAG3 expression (80). LAG3^+^ Breg cells also regulate the immunosuppressive tumor microenvironment through Bruton tyrosine kinase- and B-cell receptor-mediated signaling (81). Similar to LAG3^+^ Breg cells, LAG3 expression on B cells is induced by T cells (82). Most studies of LAG3^+^ B cells have been conducted in the context of cancer. B cell subsets expressing PD-1, TIM-3, and LAG3 have been identified in B16F10 melanoma. However, deletion of LAG3 did not enhance effector T-cell responses (83). In triple-negative breast cancer, although CD19^+^ LAG-3 ^+^ B cells comprised a minor fraction (<3%) of total B cells, most of these cells displayed high immunoglobulin D expression, suggesting that LAG3 may serve as an activation marker for B cells (84).

### 3.2 LAG3 on innate immune cells

#### 3.2.1 Dendritic cell subpopulations

LAG3^+^ plasmacytoid dendritic cells (pDCs) induce abnormal immune activation, thereby promoting inflammation (85). A previous study showed that LAG3 is expressed on CD11c^low^B220^+^PDCA-1^+^ pDCs and contributes to the homeostasis of these cells. Moreover, T cell expansion is enhanced in the presence of LAG3^-/-^ pDCs (86). More recently, it has been reported that bone marrow-derived DCs upregulate tumor necrosis factor-α (TNF-α) secretion, enhance glycolysis, and reduce fatty acid utilization for mitochondrial respiration, suggesting that LAG3 regulates the metabolic programming of DCs (87). A novel bispecific antibody targeting LAG3 and programmed death-ligand 1 has been shown to promote DC activation and elicit effective CD8^+^ T cell responses (88). In addition, Ag-specific CD49b^+^LAG3^+^ T cells, characterized by a Tr1 cell gene signature, were induced by tolerogenic IL-10-engineered DCs (DC^IL-10/Ag^). Administration of DC^IL-10/Ag^ blocked the development of type 1 diabetes (T1D) in preclinical models (89). Collectively, these findings indicate that LAG3 plays a pivotal role in regulating DC function.

#### 3.2.2 Other innate immune cells

LAG3 is also expressed on other innate immune cells and interacts with these cells. Indeed, the LAG3 locus is adjacent to the NK gene complex; however, LAG3 does not participate in a specific model of natural killing in human NK cells (90). Additionally, LAG3 expression on activated CD1d-restricted natural killer T (NKT) cells reduces NKT cell proliferation (91). It has also been demonstrated that elevated LAG3 expression is associated with decreased IFN-γ production in invariant NKT cells during chronic HIV infection (92). An *in vitro* study showed that LAG3 impairs the differentiation of monocytes into macrophages in the presence of granulocyte-macrophage colony-stimulating factor (GM-CSF), as well as their differentiation into DCs in the presence of GM-CSF and IL-4 (93). Moreover, LAG3 mediates contact-dependent inhibition between Tregs and CX3CR1^+^ macrophages, thereby suppressing the production of IL-23 and IL-1β by macrophages (75).

Taken together, LAG3 is widely expressed on a variety of immune cells and plays a central role in immune regulation, thereby forming a complex immunoregulatory network. **Figure 2** summarizes the LAG3-mediated immunomodulatory network involving immune cells.

## 4 Role of LAG3 in autoimmune diseases

### 4.1 LAG3 in autoimmune diabetes

LAG3 plays an important role in the pathogenesis of autoimmune diabetes. Genetic deletion or blockade of LAG3 exacerbates T1D in non-obese diabetic mice by accelerating the infiltration of autoreactive CD4^+^ and CD8^+^ T cells into the pancreatic islets (94). In addition, LAG3 restricts the proliferation and function of Tregs at inflammatory sites, which also contributes to the development of T1D (73). Recently, it has been reported that patients with T1D who exhibit an enriched exhausted phenotype, including increased LAG3 expression, on CD8^+^ T cells, experience slower disease progression and prolonged preservation of C-peptide levels (95). CD8^+^ T cell-specific deletion of the inhibitory receptor LAG3 disrupted this “restrained” phenotype and thereby accelerated disease progression (96). Furthermore, although LAG3 is expressed on IL-21^+^CD4^+^ T cells, these cells still accumulate in the pancreatic islets and target pancreatic β cells, contributing to the development of T1D (97). These findings support the view that the pathogenesis of AIDs, including T1D, is highly complex and that LAG3 represents only one component of the underlying regulatory network. Distinct microenvironments, diverse stimulatory factors, and multiple molecular pathways interact and cross-regulate one another, ultimately leading to disease development.

### 4.2 LAG3 in systemic lupus erythematosus

Systemic lupus erythematosus (SLE) is a prototypical autoimmune disease characterized by multiorgan inflammation driven by autoantibodies. Current research on LAG3 in SLE has mainly focused on T cells. T cell infiltration has been detected in the kidneys of various lupus mouse models. These T cells express multiple inhibitory receptors, including LAG3, and exhibit marked dysfunction characterized by reduced cytokine production and impaired proliferative capacity. This hypofunctional state is directly associated with mitochondrial dysfunction (98). As a critical coinhibitory receptor, LAG3 cooperates with PD-1 to regulate T cell exhaustion in the kidneys of SLE mice, and this serves as a compensatory mechanism to counteract autoimmune injury. Whether these LAG3-expressing exhausted T cells exert pro-inflammatory effects analogous to exhausted T cells observed in psoriasis remains to be verified by further research (99). In patients with SLE, the frequency of LAG3^+^ Tregs is decreased, and LAG3^+^ Tregs expressing transforming growth factor-β3 (TGF-β3) inhibit antibody production (79). Similar findings have been observed in lupus mouse models, where LAG3^+^ Tregs regulate B cell responses through early growth response-dependent TGF-β3 production (100). Further studies have demonstrated that Foxp3 and RORC mRNA are co-expressed in LAG3^+^ Tregs in the peripheral blood of patients with SLE; however, these cells lack suppressive capacity and secrete IL-17 (101). With advances in research on LAG3 ligands, the FGL1-LAG3 signaling axis has been shown to contribute to the pathogenesis of SLE by reducing IL-10 production by Treg cells while promoting T cell-derived IL-17A and IL-21 production (102). Notably, these dysfunctional LAG3^+^ Tregs lose partial immunosuppressive capacity under inflammatory conditions. Instead, they facilitate the proliferation of pathogenic immune cells such as Th17, ultimately triggering autoimmune-mediated tissue damage. In addition, the association between LAG3 gene expression and SLE further supports the important role of LAG3 in this disease (103, 104).

### 4.3 LAG3 in rheumatoid arthritis

sLAG3 levels are elevated in the plasma and synovial fluid of patients with RA (23). High sLAG3 levels are associated with autoantibody seropositivity in early RA, and LAG3 exerts a direct immunomodulatory role in chronic RA by reducing inflammatory cytokine production (105). Increased LAG3 expression in the synovial fluid of patients with RA is associated with multiple cytokines, indicating the complexity of immune responses in autoimmune pathology (106). The frequency of LAG3^+^ Tregs is lower in patients with RA, particularly in those with higher Clinical Disease Activity Index scores. Moreover, these LAG3^+^ Tregs inhibit B-cell antibody production (107). *In vitro*, LAG3^+^ Tregs induced by B (LAG3^+^ Treg-of -B) cells inhibit CD4^+^ T-cell proliferation through LAG3-dependent mechanism and IL-10 secretion. In the CIA model, adoptive transfer of LAG3^+^ Treg-of-B cells alleviated disease severity and reduced inflammation both locally and systemically (108). Notably, the frequency of LAG3^+^ B cells is also increased in patients with RA, and is negatively correlated with the number of tender joints and the Disease Activity Score in 28 joints using erythrocyte sedimentation rate (DAS28-ESR) (109). Increased LAG3 expression following RA treatment further supports the important role of LAG3 in RA. Clinically, glucocorticoids and tumor necrosis factor (TNF) inhibitors can upregulate CD14^+^LAG3^+^ cells, thereby exerting therapeutic effects on synovitis (110). Abatacept treatment increases the frequency of LAG3^+^ Tregs in the peripheral blood of patients with RA (111). In the CIA mouse model, FGL1, a ligand of LAG3, specifically binds to the LAG3 receptor on the surface of 3T3-LAG3 cells, thereby further suppressing the secretion of IL-2 and IFN-γ by activated primary mouse T cells. In addition, FGL1 reduces inflammatory cytokine levels in local paw tissue, prevents joint inflammation, inflammatory cell infiltration, and bone deformities, and slows the progression of CIA (35).

### 4.4 LAG3 in multiple sclerosis

Early findings regarding the association between *LAG3* gene variants and the risk of multiple sclerosis (MS) have been inconsistent. In a study of the Nordic population, germline variants of the *LAG3* gene (rs19922452, rs951818, and rs870849) were reported to confer susceptibility to MS (112). However, a subsequent replication study failed to validate these findings (113). Similarly, a study of a Caucasian Spanish population found no association between the genotypic or allelic variants of *LAG3* rs870849 and MS risk (114). More recently, a novel germline variant of the *LAG3* gene (rs2365095) was reported to be genetically associated with MS (115). Collectively, these findings support a potential association between LAG3 and susceptibility to MS. In preclinical models, Lag3^−/−^ mice developed severe experimental autoimmune encephalomyelitis (EAE). *In vivo*, IL-27 alleviated autoimmune neuroinflammation through a Treg/LAG3-dependent but IL-10-independent mechanism (116). In patients with relapsing-remitting multiple sclerosis (RRMS), the frequency of LAG3^+^ T cells is significantly reduced. Moreover, T cells from patients with RRMS exhibit global dysregulation of LAG3 expression, characterized by decreased transcription that persists even after T cell stimulation (117). Notably, LAG3 is implicated not only in the pathogenesis of MS but also in its clinical prognosis. Studies have demonstrated that reduced LAG3 expression in peripheral blood mononuclear cells (PBMCs) at the time of MS diagnosis predicts poor clinical outcomes (118).

### 4.5 LAG3 in inflammatory bowel disease

As previously mentioned, LAG3 serves as a critical mediator of the inhibitory function in of Tregs during intestinal inflammatory responses (75, 119). Molecular switches driven by Notch/STAT3 signaling and dependent on Blimp-1/c-Maf induce IL-10 expression in human CD4^+^ T cells, along with the concomitant upregulation of coinhibitory receptors, including LAG3. These anti-inflammatory pathways are impaired in patients with inflammatory bowel disease (IBD), highlighting the importance of LAG3 in IBD pathogenesis (120). A previous study showed that LAG3^+^ lymphocytes are significantly increased in the inflamed mucosal tissues of patients with ulcerative colitis and that both the frequency of LAG3^+^ T cells and LAG3 transcript levels are associated with endoscopic disease severity (7, 121). Moreover, IBD unresponsive to biological agents is associated with increased LAG3 expression on T cells (122). Interestingly, high LAG3 expression is associated with an increased risk of medium- to long-term clinical recurrence in patients with IBD (123).

### 4.6 LAG3 in psoriasis

In patients with psoriasis, LAG3 is expressed on Tregs and Tr1 cells (124). The frequencies of CD4^+^LAG3^+^ T cells (125) and Tr1 cells (126) are decreased in patients with active psoriasis, and disease activity is positively correlated with their frequencies. Gong et al. (127) found that psoriasis is associated with reduced LAG3 expression in CD4^+^ T cells and Tregs. Meanwhile, LAG3downregulation is associated with ADAM10/17-mediated LAG3 cleavage (127). Anti-TNF-α therapy has been shown to increase LAG3 expression, as well as the expression of the previously mentioned immune checkpoints TIGIT and CTLA-4 (128). In patients with psoriatic arthritis, glucocorticoids and antirheumatic drugs exert therapeutic effects by upregulating CD14^+^LAG3^+^ cells in synovial tissue and PBMCs (110). In recent years, a therapeutic strategy has been proposed to selectively deplete pathogenic T cells activated through LAG3 for the treatment of psoriasis (99). Relevant studies are discussed in the following sections.

**Figure 3:**
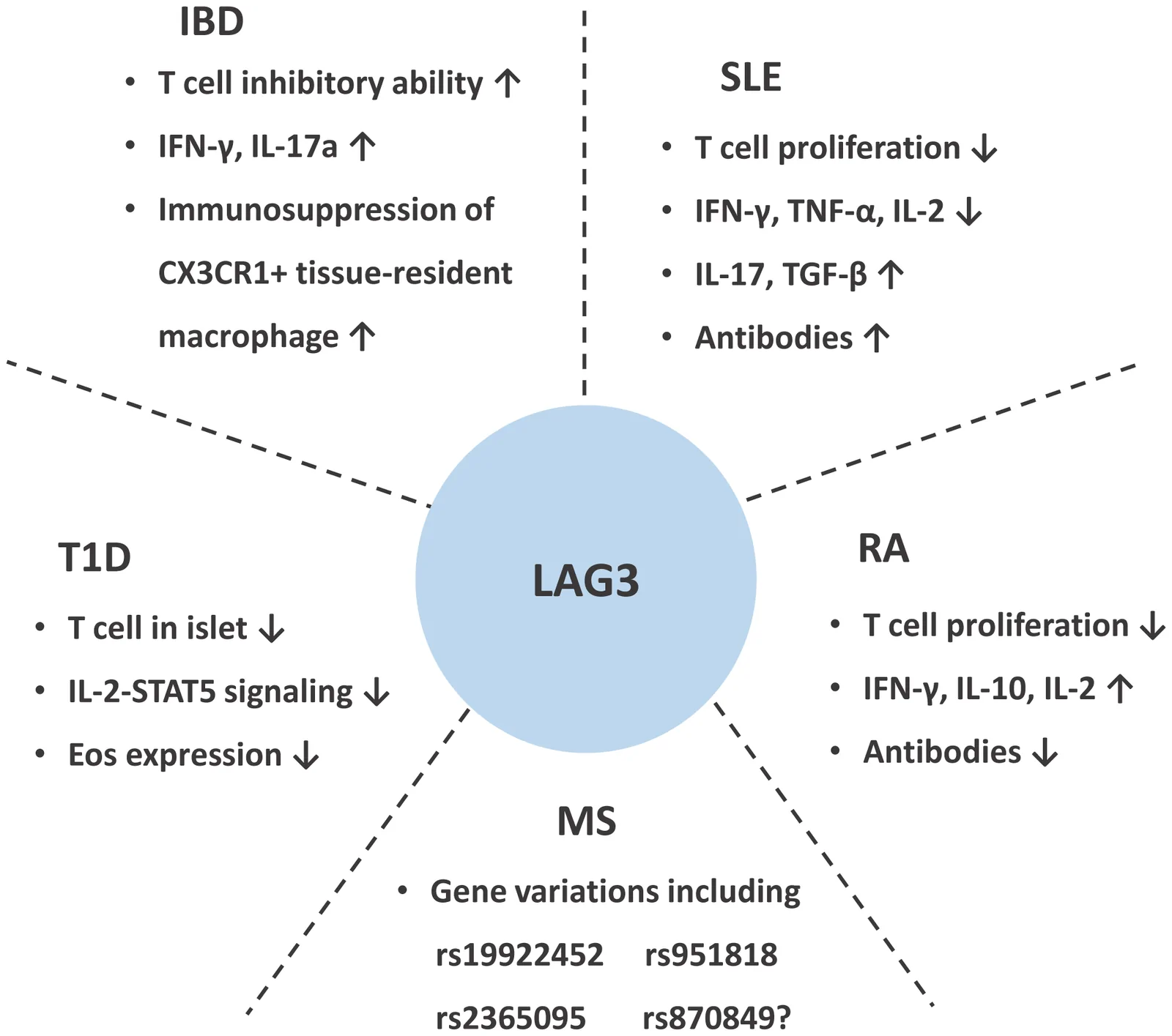
The core position of LAG3 in various autoimmune diseases. LAG3 is responsible for decreasing the proliferation and increasing the inhibitory ability of T cells. LAG3 promotes the occurrence of various autoimmune diseases by up-regulating or down-regulating the secreting of cytokines. In RA and SLE, LAG3 is involved in the pathogenesis by promoting the production of antibodies by B cells. In IBD, the engagement of LAG-3 on MHC class II drove profound immunosuppression of CX_3_CR_1_^+^ tissue-resident macrophages. MS is related to LAG3 gene variations.

**Figure 3** summarizes the mechanisms by which LAG3 is involved in several common AIDs. In addition to the diseases discussed above, only a limited number of studies have investigated the association between LAG3 and other AIDs. In ITP the T allele of *LAG3* rs870849 serves as a protective factor against severe ITP (129). A start codon variant in the *LAG3* gene (rs781745126) is associated with reduced LAG3 expression and an increased risk of autoimmune thyroid disease (130). Furthermore, patients with active thyroid eye disease exhibit elevated sLAG3 levels (131).

## 5 The therapeutic potential of targeting LAG3 in autoimmune diseases

To date, at least 19 novel molecules and biologics targeting LAG3 are at different stages of preclinical and clinical development. These agents can be broadly classified into five categories: anti-LAG3 blocking mAbs, antagonistic bispecific antibodies, anti-LAG3 depleting mAbs, LAG3 recombinant fusion proteins and LAG3 agonistic mAbs (**Table 2**). Most of these agents have been developed for cancer treatment and have demonstrated encouraging outcomes. Given the key regulatory role of LAG3 in autoimmunity, including the inhibition of T cell proliferation and function and the enhancement of Treg-cell function, LAG3 is considered a promising therapeutic target for inflammatory and AIDs. Therefore, therapeutic strategies such as LAG3 agonists and the selective depletion of LAG-3^+^ autoreactive T cells while preserving LAG-3^+^ Tregs may represent promising approaches for the treatment of AIDs.

**Table 2:** The clinical landscape of LAG3 targeted therapy for autoimmune diseases.

Type	Isotype	Representative agents	Condition or disease
Anti-LAG3 blocking mAbs	IgG4	Fianlimab	Tumor
		Favezelimab	
		Ieramilimab	
		TSR-033	
	IgG4κ	Relatlimab	Tumor
		Tebotelimab	
	IgG1	FS118	Tumor
	IgG1κ	INCAGN02385	Tumor
	IgG	IBI110	Tumor
		HLX26	
Antagonistic bispecific antibodies	Bispecific anti-LAG3/PDL1	RO7247669	Tumor
		ABL501	
		IBI323	
	Bispecific anti-LAG3/PD1	EMB-02	Tumor
	Bispecific anti-LAG3/CTLA4	XmAb22841	Tumor
Anti-LAG3 depleting mAbs	IgG1	GSK2831781	Psoriasis
			IBD
			Healthy volunteers
Agonistic mAbs	IgG4	IMP761	DTH model in the cynomolgus macaque

The rationale underling the development of LAG3 agonists is to suppress autoimmunity by inhibiting activated T cells while preserving the immune system’s ability to defend against pathogen invasion. IMP761 is a LAG3-specific humanized agonistic antibody with immunosuppressive properties both *in vitro* and *in vivo*, as demonstrated in an Ag-specific delayed-type hypersensitivity model in cynomolgus macaques (13). IMP761 exerts its therapeutic effects by inhibiting TCR-mediated nuclear factor of activated T cells activation, as well as Ag-induced T-cell proliferation and activation. This study demonstrated that triggering LAG3-mediated inhibitory immune checkpoint signaling through agonistic antibodies represents a promising and more targeted therapeutic strategy for AIDs. Currently, a clinical trial (NCT06637865) evaluating the safety of IMP761 in healthy individuals is ongoing.

GSK2831781, an anti-LAG3 depleting mAb, is a humanized antibody-dependent cellular cytotoxicity enhanced mAb designed to eliminate LAG3-positive cells in AIDs. In psoriasis, GSK2831781 reduces LAG3^+^ cell counts in peripheral blood and lesional skin, while simultaneously downregulating proinflammatory genes (IL-17A, IL-17F, IFN-γ and S100A12), and upregulating CDHR1, a gene involved in epithelial barrier integrity (99). In healthy individuals, GSK2831781 depletes circulating LAG3^+^ T cells, has no significant impact on circulating Tregs, and exhibits good tolerability (132). However, in IBD, despite evidence of LAG3^+^ cell depletion in the blood following GSK2831781 treatment, the antibody failed to alleviate colonic mucosal inflammation, suggesting limited pharmacological efficacy (133). Therefore, LAG3 agonists and anti-LAG3 depleting mAbs remain far from becoming viable targeted therapies for AIDs. The development of optimal LAG3 agonists requires elucidating the precise mechanisms by which LAG3 exerts its inhibitory effects, as well as clarifying the pathogenic mechanisms underlying various AIDs.

## 6 Conclusions

LAG3 is a key regulator of immune homeostasis and serves as a next-generation immune checkpoint. In cancer, LAG3 has emerged as the third effective immune checkpoint target. Nevertheless, the prominent role of LAG3 in maintaining immune tolerance has led to a growing number of studies investigating its involvement in autoimmune diseases. The therapeutic effects of anti-LAG3 depleting mAbs and LAG3 agonistic mAbs in psoriasis and IBD provide new insights into therapeutic strategies targeting LAG3 for AIDs. In conclusion, although LAG3 plays a crucial role in immune regulation, its molecular mechanisms remain incompletely understood, and the interactions between LAG3 and its ligands are highly complex, requiring further in-depth investigation.
